# Understanding the Effects and Adverse Reactions of Deep Brain Stimulation: Is It Time for a Paradigm Shift Toward a Focus on Heterogenous Biophysical Tissue Properties Instead of Electrode Design Only?

**DOI:** 10.3389/fnhum.2018.00468

**Published:** 2018-11-27

**Authors:** Christian Ineichen, Naomi Ruth Shepherd, Oǧuzkan Sürücü

**Affiliations:** ^1^Department of Psychiatry, Psychotherapy and Psychosomatics, Psychiatric University Hospital Zurich, Zurich, Switzerland; ^2^Institute of Biomedical Ethics and History of Medicine, University of Zurich, Zurich, Switzerland; ^3^Center for Psychiatry Emmendingen, Emmendingen, Germany

**Keywords:** deep brain stimulation, finite element method, subthalamic nucleus, field modeling, biophysical properties, conductivity, permittivity, permeability

## Abstract

Deep brain stimulation (DBS) has been proven to be an effective treatment modality for various late-stage neurological and psychiatric disorders. However, knowledge on the electrical field distribution in the brain tissue is still scarce. Most recent attempts to understand electric field spread were primarily focused on the effect of different electrodes on rather simple tissue models. The influence of microanatomic, biophysical tissue properties in particular has not been investigated in depth. Ethical concerns restrict thorough research on field distribution in human *in vivo* brain tissue. By means of a simplified model, we investigated the electric field distribution in a broader area of the subthalamic nucleus (STN). Pivotal biophysical parameters including conductivity, permittivity and permeability of brain tissue were incorporated in the model. A brain tissue model was created with the finite element method (FEM). Stimulation was mimicked with parameters used for monopolar stimulation of patients suffering from Parkinson’s disease. Our results were visualized with omnidirectional and segmented electrodes. The stimulated electric field was visualized with superimpositions on a stereotactic atlas (Morel). Owing to the effects of regional tissue properties near the stimulating electrode, marked field distortions occur. Such effects include, for example, isolating effects of heavily myelinated neighboring structures, e.g., the internal capsule. In particular, this may be illustrated through the analysis of a larger coronal area. While omnidirectional stimulation has been associated with vast current leakage, higher targeting precision was obtained with segmented electrodes. Finally, targeting was improved when the influence of microanatomic structures on the electric spread was considered. Our results confirm that lead design is not the sole influence on current spread. An omnidirectional lead configuration does not automatically result in an omnidirectional spread of current. In turn, segmented electrodes do not automatically imply an improved steering of current. Our findings may provide an explanation for side-effects secondary to current leakage. Furthermore, a possible explanation for divergent results in the comparison of the intraoperative awake patient and the postoperative setting is given. Due to the major influence of biophysical tissue properties on electric field shape, the local microanatomy should be considered for precise surgical targeting and optimal hardware implantation.

## Introduction

Deep brain stimulation is an effective treatment modality for various neurological disorders (Lozano and Lipsman, 2013). In particular, its beneficial effect has been confirmed in randomized, controlled trials for Parkinson’s disease (PD) (Deuschl et al., 2006; Weaver et al., 2009; Follett et al., 2010). The use of DBS is also increasing as therapy for psychiatric conditions (Clair et al., 2018).

In DBS, single or multiple leads create electrical fields within specific targets in the brain. These fields modulate electro-chemical networks. However, the beneficial effects may vary due to different contributing factors. These factors include: type of disease, changes in drug regimen, surgical complications, premorbid personality traits (Ineichen et al., 2016) and current leakage into the surrounding tissue, or the inability to direct the current. Precise knowledge of the biophysical interaction between stimulation and neural elements is still lacking (Kuncel and Grill, 2004). Moreover, the exact degree of electric field spread is unknown. Due to deficient knowledge on specific stimulation-effects, a good clinical outcome can only be obtained through a time-consuming adjustment of postoperative parameters (Ineichen et al., 2014). This adjustment is largely based on phenotypic observation of the patient. Such a fine-tuning is an expensive ad hoc “trial and error” process, which may be a source of discomfort and possible danger for the patient. During this adjustment, the focus typically lies on stimulation intensity (Dayal et al., 2017). However, a significant amount of research has concentrated on other parameters, such as stimulation frequency (for investigations on the beta and gamma frequency range, see e.g., Jenkinson and Brown, 2011; Tinkhauser et al., 2017; Lofredi et al., 2018). While a straightforward calculation of the electric field expansion is possible when based on stimulus intensity and frequency, a much greater challenge is presented by the incorporation of the biophysical properties of anisotropic anatomical tissue (e.g., McIntyre et al., 2004; Miocinovic et al., 2006, 2009). Side effects of DBS are frequently observed when the electrical field reaches unintended brain areas, such as the corticospinal or corticobulbar tracts. This problem is most commonly observed in patients suffering from PD, especially if the STN, a region with relatively high anisotropy and inhomogeneity (Shimony et al., 1999), has been targeted. It is often impossible to explain these side effects from an anatomical perspective when physicians refer to simple ellipsoid or spherical geometrical models around the stimulated contact.

Present technical developments addressing the drawback of current leakage have led to a change of lead configuration. Among other developments, segmented electrodes have been introduced. The contacts of segmented electrodes are split along their circumference, thus allowing a steering of the electrical field in a predefined horizontal direction (e.g., Schüpbach et al., 2017). Initial studies using segmented electrodes concluded that current steering enables symptom-specific optimization of DBS parameters. This improvement also results in a reduction of the programming burden (e.g., Contarino et al., 2014; Pollo et al., 2014; Bour et al., 2015; Fernández-García et al., 2017). Furthermore, the therapeutic window is increased (Dayal et al., 2017). These studies have also demonstrated that the direction of stimulation correlates with the adverse effects generated by unintended fiber-tract activation. Common side-effects during lateral stimulation in STN-DBS include focal muscle contraction and dysarthria as a result of corticobulbar tract activation (Dayal et al., 2017). Pyramidal tract activation has been shown to occur at stimulation thresholds used in clinical settings and compromises increase in stimulation strengths because side effects, such as speech disturbances, may be provoked (Mahlknecht et al., 2017). In a feasibility study all patients remained on directional stimulation with no back-switching to conventional omnidirectional stimulation (Steigerwald et al., 2016). Simultaneously, many theoretical considerations assume the electric field to be spherical or ellipsoid, depending on the type of stimulation (monopolar vs. bipolar) and the choice of active contacts [narrow- and wide-stimulation configuration (Montgomery, 2010)]. This assumption only holds true in a perfectly homogenous tissue. In the case of commonly used monopolar stimulation many investigators and sales representatives were led to assume a homogenous symmetrical field expansion. However, the biophysical properties of the tissue in the vicinity of the stimulating electrode are neither homogenous nor lacking influence (e.g., McIntyre et al., 2004; Butson et al., 2006, 2007; Miocinovic et al., 2006, 2009; Sotiropoulos and Steinmetz, 2007). Accordingly, these authors have already outlined that brain tissue anisotropy and inhomogeneity directly impact the field distribution. This is especially apparent since these tissue differences are even observed on a macroscopic level with Klingler’s fiber dissection method (Silva and Andrade, 2016). Due to the difficulties in measuring or visualizing the electric field in brain tissue (Wårdell et al., 2015), the anatomical structures that are reached by the electric field remain elusive. This uncertainty increases the challenge of optimal selection of stimulation parameters. Furthermore, the human subcortex is a highly populated area, where only 7% of the individual structures are depicted in standard MRI atlases (Forstmann et al., 2017), so that visual aids are of limited help. Owing to its anatomical intricacy, MRI of the subcortex only lends support by using high-field MRI (3 T or higher). Hence, because of imprecise electrode placement (e.g., Gilmore et al., 2017) due to the use of preoperative low field structural MRI images, electrode placement planning may lead to unknown electrical fields in brain tissue and hamper an optimal clinical outcome. Meanwhile, computational models have supported the advance of DBS technology (e.g., Butson et al., 2006; Miocinovic et al., 2006; Gunalan et al., 2017). Notwithstanding their importance, computational models investigating the effects of biophysical properties on current spread have yet to provide more precise and applicable knowledge on voltage distribution. While some models, in an attempt to reduce complexity, assume a cubic or cylindrical volume of rather homogenous brain tissue (e.g., Hemm et al., 2005; Buhlmann et al., 2011; Howell et al., 2015), others attempted to implement the complexities that are inherent in tissue anisotropy and inhomogeneity (e.g., Åström et al., 2006; Butson et al., 2007; Sotiropoulos and Steinmetz, 2007; Yousif et al., 2007; Miocinovic et al., 2009). Thorough and direct *in-vivo* investigation of the electric field spread in human brain tissue is not feasible and the topic has, unfortunately, not yet been addressed in depth in animal studies (for exceptions, see e.g., Miocinovic et al., 2006 or Martens et al., 2011). Therefore, theoretical models that account for influences of biophysical properties near the stimulation site are still needed. The aim of this work is to increase awareness of this need. In order to strengthen our argument, we attempt to provide a simplified simulation of the electric field expansion that (1) depicts the current distribution along a larger area of tissue, (2) includes some of the realistic tissue properties that influence the area that will be reached by DBS within and near the STN and (3) compares the effects of omnidirectional to segmented lead designs. It is our hope that this work will spark discussions and provide impetus for further investigation of this topic.

## Materials and Methods

### Introduction

When an electric potential is delivered through an implanted electrode contact, the electrically charged particles produce an electric field. This field exerts a force on other electrically charged objects. The electric field is commonly described as the force per unit charge that would be experienced by a stationary point charge at a given location in the field. The direction of the electric field is equal to the direction of the force that would be exerted on a positively-charged particle. The electric field is also defined as the negative rate of change of the electric potential. It may therefore be described by measuring the electric potential in different locations.

The electric field distribution around the electrode depends on the shape, impedance of the electrode (see e.g., Wei and Grill, 2005) and the conductivity of the tissue. Generally, electromagnetic fields propagate with a finite velocity. In turn, this finite velocity depends on the permittivity and the permeability of the brain tissue. Both, the tissue inhomogeneity and anisotropy in the vicinity of the electrode, can alter the shape of the electric field. Especially in the case of the STN-region the tissue heterogeneity is high (Shimony et al., 1999). It is therefore difficult to mimic the details of various cell shapes, cell distribution and extracellular properties. On a macroscopic level, a material is described as having electric permittivity and conductivity. The permittivity describes the ability of the material to store charge, while the conductivity describes the ability to conduct electric currents.

Since various factors influence the field distribution in DBS, many simulation parameters such as waveform shape, frequency, pulse width and amplitude, and electrical properties of brain tissues have to be included.

### Theoretical Model

The finite element method (FEM) was used for our study. FEM is a widely applied numerical technique used to calculate approximate solutions of general partial differential equations (PDEs) and integral equations for different problems in physics. FEM has been applied previously in the context of DBS (e.g., Buhlmann et al., 2011; Pollo et al., 2014; Howell et al., 2015). PDEs describe physical problems, which are considered over a certain area. Instead of seeking an approximation to the problem that applies to the entire area, the basic idea of FEM is to subdivide the complex anatomical area into smaller mesh elements with simple shapes. Common mesh elements may have triangular, square, tetrahedral, or cubical shapes. A simplified approximation to the problem can be attained for each mesh element. Thus, the first step to solve a problem with the FEM is to subdivide the area into a finite number of mesh elements. Then the type of applied approximation is chosen for the individual elements. This approximation is called a shape function or base function and is normally a polynomial of linear, quadratic or cubical degree. The shape functions are formulated according to the type of mesh elements that are used and the physics that is to be solved. For this work, the commercially available software tool Comsol Multiphysics (Comsol AB, Sweden) was used to implement and solve finite element models.

### Conductivity

Briefly put, conductivity (σ) is a property of a material and allows for charged particles to flow in a direction defined by an electric field. Hence, conductivity is a measure of a material’s ability to conduct an electric current. Conductivity, therefore, is the reciprocal of electrical resistivity. Metal, for example, has a higher conductivity than pure water. Conductivity is calculated by measuring the ratio of the current density to the electric strength (Siemens per meter, S/m). The incorporation of conductivity parameters into the model is important because an electric field will rearrange the electrically charged ions of a material in such a way that they will seek the lowest energy state and cancel out the electric field. The higher the conductivity, the faster and more complete this cancellation will be. If ions collide during this process, electromagnetic energy will dissipate as a result of collisions and be turned into heat.

The conductivity of a material is defined as the ratio of the current density *J* to the electric field strength *E*:

$$\sigma =\frac{J}{E}[{\mathrm{Sm}}^{-1}]$$

**FIGURE 1:**
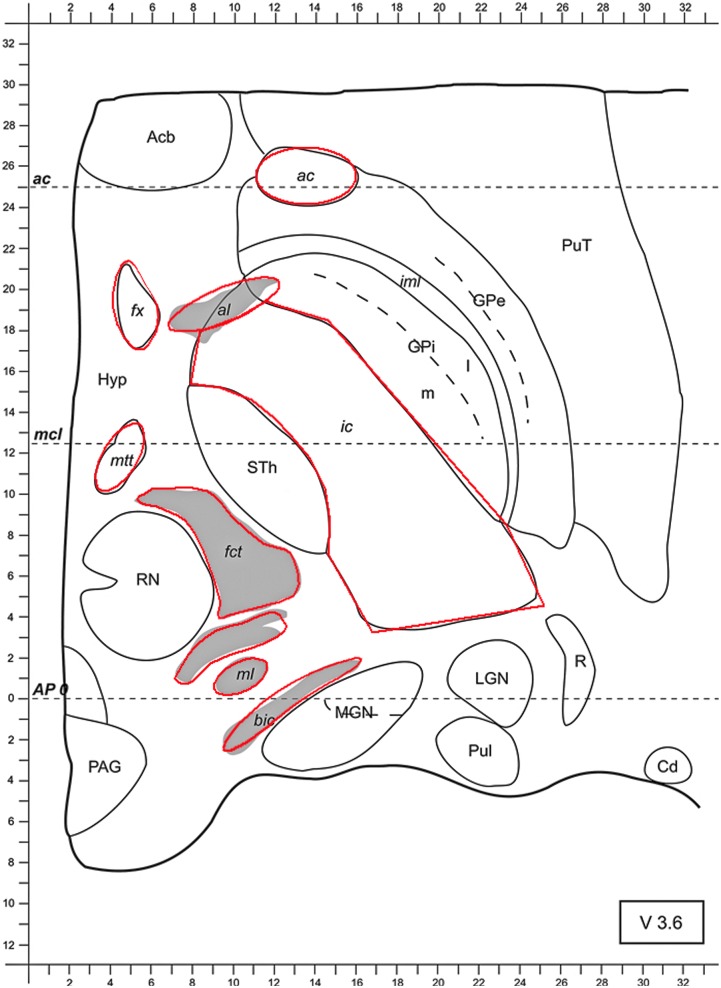
Axial slice from the stereotactic atlas of Morel 3.6 mm ventrally to the AC-PC-level (anterior commissure-posterior commissure). At approximately similar levels in patients the STN can be targeted along a parallel line to the *x*-axis at the anterior border of the RN. Conductivity values were set to 1.0 S/m for ac, fx, al, ic, mtt, fct, and ml. Bic was attributed with a conductivity of 0.1 S/m, whereas gray matter was attributed with a conductivity of 0.2 S/m. Abbreviations: anterior commissure (ac), fornix (fx), ansa lenticularis (al), internal capsule (ic), mammillothalamic tract (mtt), fasciculus cerebello-thalamicus (fct), medial lemniscus (ml), brachium of the inferior colliculus (bic), medial and lateral geniculate nucleus (MGN and LGN, respectively), pulvinar (Pul), reticular thalamic nucleus (R), globus pallidus internus and externus (GPi and GPe, respectively), putamen (Put), nucleus accumbens (Acb), hypothalamus (Hyp), subthalamic nucleus (STh; the subthalamic nucleus is abbreviated with Sth, whereas the abbreviation STN is more commonly used), red nucleus (RN), periaqueductal (or central) gray (PAG), caudate nucleus (Cd).

To this end and due to the anisotropic fiber-tract characteristics of white matter, conductivity values of 1.0 S/m were used for parallel, myelinated fibers. These include: the ac, the fx, the al, the ic, the mtt, the fct and the ml (see Figure 1). Conductivity values of 0.1 S/m were used for the perpendicular fiber bundle termed the bic (Nicholson, 1965; Gabriel et al., 1996; Kuncel and Grill, 2004). To simplify the model, the rest of the tissue of the axial slice, i.e., the isotropic central nervous system (CNS), gray matter such as the MGN, LGN, the pulvinar (Pul), the reticular thalamic nucleus (R), the GPi, GPe, the putamen (Put), the Acb, the Hyp, the STh, or STN, the RN, the periaqueductal (or central) gray (PAG) and the Cd, was attributed with a uniform, average conductivity of 0.2 S/m (Kuncel and Grill, 2004) (see Figure 1). The chosen range of the used conductivity values corresponds to a more recent publication [Koessler et al. (2017)—white matter and gray matter conductivity: 0.17 and 0.26 S/m, respectively].

### Permittivity and Permeability

Permittivity as a measure of how an electric field affects and is affected by a medium and permeability as the magnetic equivalent of permittivity were also incorporated in our model. More precisely, permittivity is a measure of the degree of polarization within the medium due to the electric field and the ensuing reduction of the electric field inside the material. In turn, permeability is a measure of the ability of a material to support the formation of a magnetic field, in other words the degree of magnetization that a material obtains in response to an applied magnetic field.

In our investigation, a static approximation is used for simulation of the electric field. Moreover, a static approximation may even be used for simulation with altering currents.

As a value of permittivity 1.05 × 10^2^ F/m (farads per meter) were used. The influence of permeability was set at 1.25 × 10^-6^ N × A^-2^ or H/m (henries per meter).

### Modeling DBS Parameters

For the simulations, the following parameter settings (in clinical use) were applied. Frequency was set to 130 Hz (hertz) in the form of rectangular pulses. A pulse width of 60 μs (microseconds) and a current intensity of 2 mA (milliamperes) were chosen in case of omnidirectional electrode configuration. In order to simulate the monopolar omnidirectional electrode configurations, the active electrode contact was set to cathode and the outer boundaries of the model to anode. In order not to influence the results, the outer boundaries were placed at a sufficient distance. Regarding the tripartite, segmented electrode configuration, 2/3 mA was used in order to obtain more comparable results (see below). Moreover, frequency was set to 130 Hz in the form of rectangular pulses of two segments with opposite current direction at a pulse width of 60 μs each. Concerning the positioning of the electrodes, we used the commonly targeted part of the sensorimotor (superior-lateral) functional zone of the STN. This area has been shown to be an optimal target for DBS in PD (Coenen et al., 2008; Horn et al., 2017). Regarding the segmented electrode, and because multiple active poles have not led to an increase in the therapeutic window compared to stimulation with the best pole alone (Pollo et al., 2014), the single active element was positioned medially, as proposed by Pollo et al. (2014).

### Visualization

For our modeling approach, axial slices from the multiarchitectonic and stereotactic atlas of the human thalamus were used (Morel, 2007). We investigated the effect of unilateral stimulation on the electric field distribution. The current lack of models was accommodated by use of axial slices of the human thalamus and the inclusion of more realistic shapes of the stimulating and surrounding structures.

### Governing Equation and Boundary Conditions

The distribution of the electric field in the vicinity of the electrode was calculated with the equation of continuity for steady currents. A trivial interpretation of the equation of continuity for steady currents is that the total amount of current within a region can only change by the amount that passes in or out of the boundary of the region. Thus, the total amount of current is preserved and cannot increase or decrease. For this reason, it can only move from one place to another.

In case of modeling the clinically relevant omnidirectional monopolar stimulation, the electrode configuration was simulated by setting the outer boundaries of the model to anode and the active electrode contacts to cathode. In order not to influence the results, the outer boundary needs to be located at a sufficient distance from the active electrode contacts. Simulations showed that already at a distance of 2 mm between the active electrode contact and the outer boundary the impact on the simulated electric field was negligible. For segmented electrodes, analogous modeling was used.

As already outlined above, FEMs were developed for simulations of the spatial distribution of the electric field. The distribution of the electric field in the vicinity of the electrode was calculated using the equation of continuity for steady currents:

$$\nabla \cdot J=-\nabla \cdot (\sigma \Delta V)=0\;[A\;m^{-3}]$$

where ∇ is the divergence, *J* (A m^-2^) the current density, σ (S m^-1^) the electrical conductivity, ∇ the gradient, and *V* (V) the electric potential.

In homogeneous and isotropic electrical conductivity, the equation of continuity for steady currents is reduced to Laplace’s equation:

$$\nabla^{2}V=0\cdot [V\;m^{-2}]$$

where ∇^2^ is the Laplace operator.

### General Models and Simulations

General finite element models (axially symmetric and 3D) were used to study the effect of the electric field when monopolar omnidirectional and one-directional, segmented DBS stimulation of the STN are applied. The basal ganglia and the anatomy of the axial slice was simplified to shapes as indicated in Figure 1. Uniform tissue was assumed for each shape.

An omnidirectional electrode with a diameter of 1.27 mm, contact length of 1.5 mm, separated by 0.5 mm (Lead Model 3389, Medtronic Inc., United States) and a segmented electrode (Vercise DBS Directional Lead Model, Boston Scientific, United States) with a lead diameter of 1.3 mm, contact length of 1.5 mm with a contact spacing (axially) of 0.5 mm, were modeled. Both electrodes were positioned in the STN 3.6 mm ventrally to the AC-PC-level. In this position the STN may usually be targeted along a parallel line to the *x*-axis at the anterior border of the RN (Bejjani et al., 2000; Rabie et al., 2016) (Figure 1). The potential values around and at the position of the electrode in the *x, y* and *z* axes, described by the solution of the Laplace equation, were written in a matrix with a spatial resolution of 0.05 mm. The *x* and *y* axes lie in the plane of V 3.6 and the *z* axis is perpendicular to these planes. The electrode shaft, which is electrically isolated, was omitted to prevent unrealistically high potential values during the calculation.

The models were solved for approximately 80,000 elements and the electric field was visualized in a color-coded way. A further increase of the number of elements did not have an observable effect on the end-results as presented by the color-coded graphs of the electric field.

## Results

The model of the electric field distribution was superimposed on an axial slice from the stereotactic atlas of Morel (2007).

**FIGURE 2:**
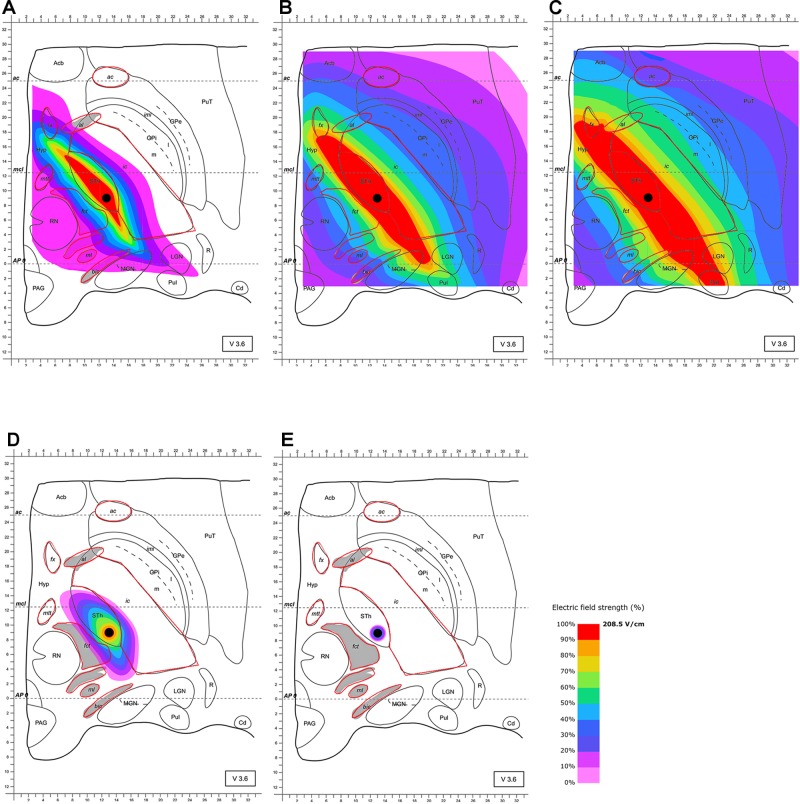
Simulation of the electric field generated by omnidirectional monopolar stimulation (130 Hz, 60 μs and 2 mA) with the electrode lead positioned in the STN (black circle within “Sth”). Electric field distribution after **(A)** 10 μs; **(B)** 40 μs; **(C)** 63 μs (maximal field extension); **(D)** 67 μs and **(E)** 68 μs; for abbreviations see Figure 1.

The stimulation of the STN with an omnidirectional electrode and commonly used clinical parameters results in a quasi-ellipsoid shape electric field distribution. Owing to the influence of heavily myelinated structures such as the ic and fct in the vicinity of the STN, the generated field is significantly distorted. Maximal field extension was detected after 63 μs (Figure 2). During the duration of the pulse, larger volumes extend asymmetrically with parts of the high current density field (100%, 208.5 V/m) stretching along the whole anterior-posterior direction predominantly affecting the whole STN. This affection includes the limbic (i.e., anterior inferior-medial) subdivision and substantial parts of the fx and the posterior, but also the anterior ic, the MGN and LGN, the Hyp and the pulvinar. Moreover, 104.25 V/m of field strength (50%) additionally reaches the Hyp, the whole ic, the mmt, the fct, parts of the ml and GPi, the bic, the al, parts of the reticular thalamic nucleus (R) and the whole pulvinar (Pul), MGN and LGN. Given the fact that Figure 2 proves an unexpected extension of electrical current to geniculate thalamic and pulvinar areas, changes in emotion perception could be attributed to impaired visual and auditory processing (Péron et al., 2010, 2013; Symons et al., 2016; Ferrara et al., 2017).

**FIGURE 3:**
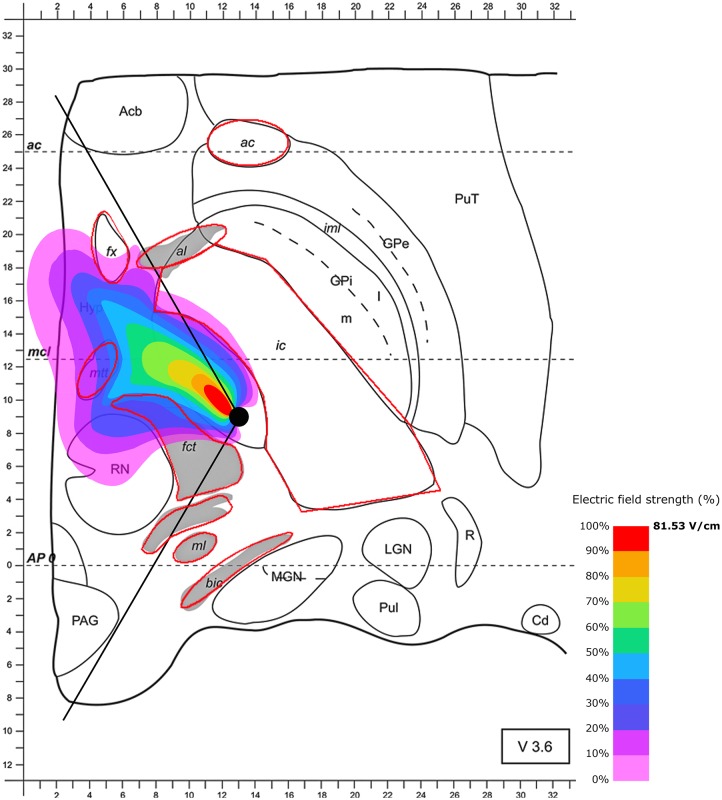
Simulation of the electric field generated by segmented electrode stimulation through one active pole (130 Hz, 60 μs and 2/3 mA) with the electrode lead positioned in the STN. Positioning of the electrode’s active pole according to Pollo et al. (2014) (medial direction of the active pole). Electric field distribution after 60 μs (maximal field extension); for abbreviations see Figure 1.

Next, we were interested in the effect of the local tissue properties on the electric spread during the use of segmented electrodes and the regions reached during stimulation. First, we modeled the segmented electrode positioned in the medial direction (as suggested by Pollo et al., 2014). The use of segmented electrodes led to less current leakage when compared to omnidirectional stimulation. Notably, targeting of parts of the posterior STN is improved when the electrical field is confined to a partly posterior and medial quadrant of the STN in the axial slice in case of maximal electric field extension after 60 μs (Figure 3). As with the omnidirectional configuration, the surrounding tissue properties influence the shape of the electric field, resulting in a cone-like distribution. The electrically stimulated area aligns along the anteromedial axis with parts of the field spilling over the electrode. In the higher magnification presented in Figure 3, the diffusion of the electrical current in the opposite direction of the active electrode pole is better visible. Notably, the sensorimotor segment of the STN defined as the posterior, superior and lateral third of the STN (Coenen et al., 2008; Plantinga et al., 2016) appears to be reached only insufficiently when this lead configuration is chosen. Additionally, current spread toward the mtt and fx may influence memory formation. Moreover, stimulation of anterior-medial and ventral parts of the STN unintendedly cause interference with limbic and associative functions.

**FIGURE 4:**
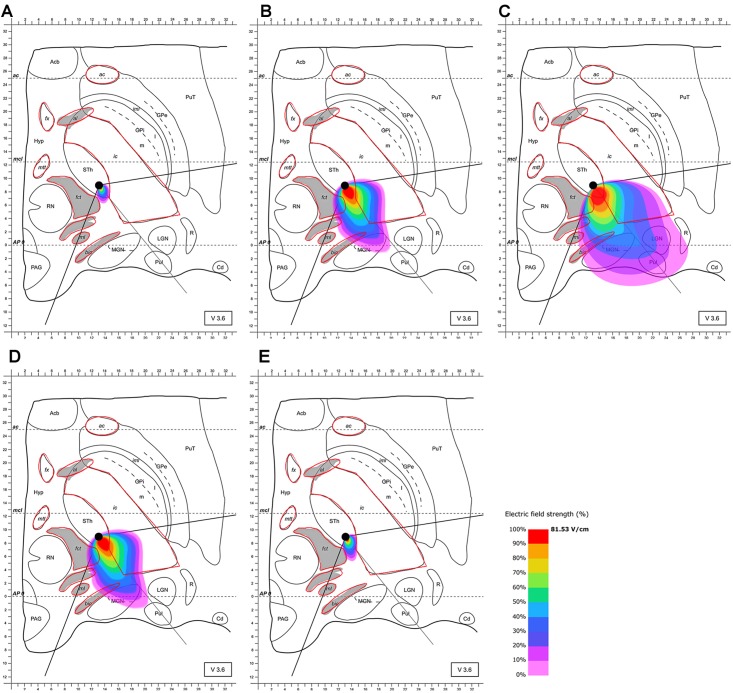
Simulation of the electric field generated by segmented electrode stimulation through one active pole (130 Hz, 60 μs and 2/3 mA) with the electrode lead rotated 128° counter-clockwise relative to the situation described in Figure 3 and positioned in the STN. Electric field distribution after **(A)** 10 μs; **(B)** 40 μs; **(C)** 60 μs (maximal field extension); **(D)** 61 μs and **(E)** 62 μs; for abbreviations see Figure 1.

**FIGURE 5:**
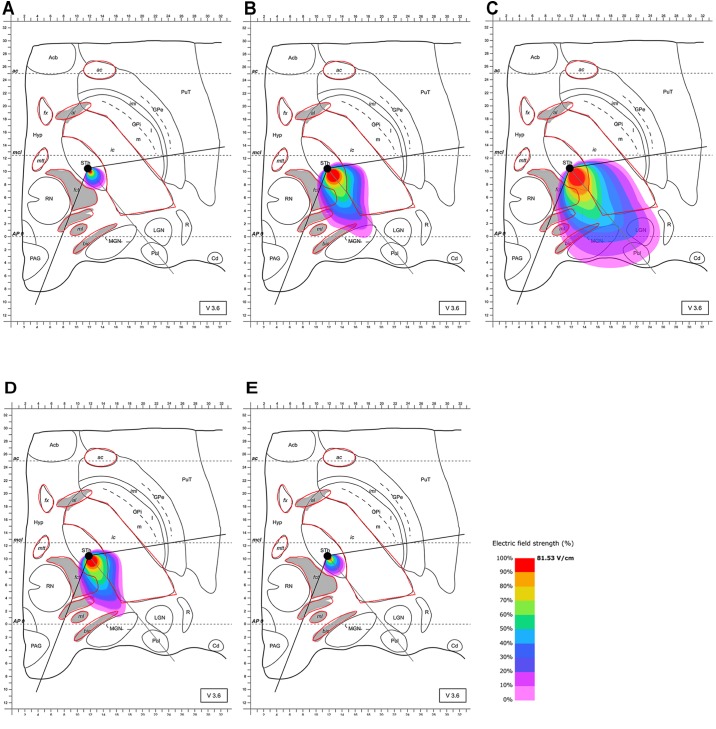
Simulation of the electric field generated by segmented electrode stimulation through one active pole (130 Hz, 60 μs and 2/3 mA) with the electrode lead rotated 128° counter-clockwise relative to the situation presented Figure 3 and positioned another 2 mm anterior along the AP-axis in the STN. Electric field distribution after **(A)** 10 μs; **(B)** 40 μs; **(C)** 61 μs (maximal field extension); **(D)** 61.5 μs; **(E)** and 62 μs; for abbreviations see Figure 1.

Based on these results and the proposed positioning of the segmented electrode according to Pollo et al. (2014), it became evident that the electrode should be rotated in the direction of the MGN and along the AP-axis that connects the edges of the almond-shaped STN in order to more accurately target the superior-lateral and posterior STN. The electrode was rotated accordingly, and the electric field distribution was simulated for this lead configuration. The electrode was set as follows: (1) at the same point as previously (see Pollo et al., 2014) but rotated by 128° counter-clockwise compared to Pollo et al.’s configuration with the active pole directed to the MGN (and the AP-axis of the STN); (2) 2 mm further anterior. This further anterior setting is consistent with selectable parameter settings of commonly used stereotactic frames (Figures 4, 5). The rationale for performing step (1) and (2) was to possibly achieve a better targeting of the posterior and lateral part of the STN. The modeling results of scenario (1) showed improved targeting of the sensorimotor STN (Figure 4). Scenario (2) indicated that the posterior-lateral quadrant of the STN is even better targeted if the electrode is placed 2 mm further anterior along the constructed AP-axis of the STN in addition to the application of scenario (1) (Figure 5, compared to Figure 4). Consistent with the narrow position of the STN between the ic and the fct, the electric field is squeezed in between the myelinated white matter tracts, whereby the posterior STN is reached with 100% of the applied energy. Current flow reaches the borders of the ic and fct with 80% of the applied energy, and parts of the postero-lateral fct and postero-medial ic are reached with 50% of the delivered energy (40.7 V/m).

## Discussion

Reliable knowledge about the electric field distribution in the brain tissue is crucial for DBS. The aim of this study was to apply a simplified model of the electric field generated by common stimulation parameters in the human brain tissue. In particular, the effects of permittivity, permeability and conductivity on field shape were considered and the influence of the latter were depicted on a larger area of brain tissue. The visualization of the electric field illustrates the distribution of the current flow in case of omnidirectional and segmented electrodes. We hope that our results will stimulate interest and raise clinicians’ awareness of the influence of the brain tissue properties on the electric field shape. While current density may be extracted from the proposed model, so far no direct link has been established between current density and the neural response to stimulation (McIntyre and Thakor, 2002; McIntyre et al., 2004). Accordingly, the following neurophysiological considerations have to be treated with caution.

### Side Effects: Adverse Neurophysiological Effects of STN-DBS Based on Modeled Current Leakage

The modeling of an adjusted segmented electrode showed a more homogenous stimulation of the sensorimotor region of the STN when compared to omnidirectional stimulation. Vast current leakage into the surrounding tissue was observed for omnidirectional stimulation when clinically used parameter settings were chosen. With this standard electrode, the activated tissue encompasses the electrode completely and the steering of neuronal tissue stimulation in a defined direction is not possible. Omnidirectional monopolar stimulation can affect the limbic subterritories of the STN, the anterior part of the ic (a DBS target for the treatment of major depressive disorder), the Hyp and mamillothalamic tract. These areas also seem to be partly affected by medially turned segmented stimulation. The mentioned structures appear to be involved in emotional processing and their stimulation may therefore lead to significant side-effects. An increase of preexisting cognitive deficits, especially regarding memory function, was observed after DBS. If certain preexisting conditions are at hand, clinicians are led to switch to Gpi instead of STN targeting (Da Cunha et al., 2015). Current expansion into the mamillothalamic or even fornical area may be responsible for the observed side-effects in STN targeting. Furthermore, paresthesia (via information traveling through the ml and spinothalamic tract that ascends posteriorly of the STN to the cerebral cortex and the posterior thalamus), disconjugate gaze and diplopia (via axons coming from the oculomotor nucleus near the RN), conjugated deviation of gaze (via projections to the frontal eye field), light sensations (phosphenes via ventral GPi stimulation including the optic tract), cognitive and emotional alterations (via anterior GPi stimulation) and tonic muscle contractions (via the corticospinal and corticobulbar tract in the ic, ventral and lateral to the thalamus) can occur due to inadvertent co-stimulation (Montgomery, 2010). Only an incomplete and brief outline of potential unwanted neurophysiological effects is given here. These effects may lead to undesired sequelae if certain regions are reached, as seen in the results of omnidirectional, conventional STN-DBS, in particular. This corroborates the need for revised and improved precision targeting in DBS.

### Recommendations and Visions: Electrode Positioning for More Effective Sensorimotor STN-DBS Based on Simulation Results

The STN is situated in the junction of the diencephalon and mesencephalon, lateral to the brachium conjunctivum, the RN and the fct, medial (and dorsal/superior) to the ic and ventral/inferior to the thalamus (Nieuwenhuys et al., 2007). Therefore, the stimulation of the STN is, as mentioned above, likely to influence non-targeted structures. The current of the generated electric field differs according to stimulation chosen (omnidirectional vs. segmented). The large field extension during monopolar stimulation is compatible to the induction of a far-field dipole between the single active contact and the case of the implanted pulse generator. Visualization of the simulated electric field confirms that biophysical characteristics of the brain tissue have an important influence on the shape of the electric field. This is particularly striking when a larger area of tissue is examined. So far, however, the influence of inhomogeneous and anisotropic tissue appears to have been largely neglected in DBS practice. This neglect evidently occurred despite the presence of insight based on even more accurate computations (see e.g., Butson et al., 2006, 2007; Sotiropoulos and Steinmetz, 2007; Miocinovic et al., 2009). Evidence for the latter statement can be drawn from the universally similar application of electrode configurations and parameter settings, which appear to be used irrespective of tissue properties at the stimulation side for various neuronal areas.

The extent to which the brain tissue is stimulated depends highly on the local conductivities. Large myelinated structures (for example the ic and the fct) have dielectric tissue properties, which operate as insulators. Such structures distort the electric field as they transform the theoretically spherical shape (for monopolar omnidirectional stimulation) into an ellipsoidal one. Owing to regional specificities, the described electric field shape only relates to the STN’s characteristic neuronal embedding and cannot be generalized to other areas. The present model and its possible further extensions, including individualized topographic mapping, may enable more precise electrode positioning. Clearly, segmented electrodes are better suited for precise current application.

The results show that the effects of biophysical properties are neglected in conventional STN stimulation. According to our model, the positioning of the lead has to be adjusted as described above in order to more accurately target the sensorimotor, postero-lateral STN. However, a more accurate stimulation of the sensorimotor STN does not simply imply a better clinical outcome. Clinical sequelae, for example, have been shown to pertain to current flow in the postero-lateral direction with an emergence of dysarthria, muscular contraction and paraesthesia (Pollo et al., 2014). A better understanding of current flow will hopefully lead to a greater appreciation of the influence of regional tissue properties. If knowledge could converge to the extent that specific fiber-tracts were stimulated for optimal clinical outcome, the electrodes and their orientation would have to be adjusted accordingly. In the future, segmented and other multi-contact electrodes should be positioned in an evidence-based fashion in which regional tissue properties are considered (for an investigation into lead design and its influence on the volume of tissue activated, see e.g., Butson and McIntyre, 2005).

More specifically, our results demonstrate that the electrode lead should be (1) rotated 128° counter-clockwise when compared to Pollo et al. (2014, see Figure 3) and (2) positioned 2 mm further anterior along the AP-axis of the STN in order to optimally stimulate its sensorimotor area. Based on results of the presented stimulation, no advantage may be derived from a tripartite-electrode design. As outlined above, multiple active poles do not appear to lead to an increase in the therapeutic window when compared to stimulation with the best pole alone (Pollo et al., 2014). In principle, however, an increase in the number of contacts should lead to increased versatility, as many optimal configurations may be attained (Howell et al., 2015). Perioperative MRI or computer tomographic (CT)-imaging to assess precise positioning of the electrode remain an ultimate goal for best DBS results (Hariz, 2017).

There is an evident danger of refraining from revisions of suboptimal placement of electrodes because of a reliance on more numerous choices for stimulation. However, present models of the electrical spread of segmented electrodes are unfortunately far too simple when it comes to confirming this threat.

The electric field shape of each individual target is characterized by its own cytoarchitectonic micro- and macro-environment. This being the case, there is no universal electrode geometry or configuration which is optimal for each DBS target (see also Howell et al., 2015). Because of the immense influence of biophysical tissue properties on the shape of the electric field, the optimal stimulation of different targets will necessitate different parameter selections, electrode types and configurations (for example with regard to rotation). Results from another project (data not shown) demonstrate that electrical stimulation near the skull expectedly leads to marked field distortions. This outcome further corroborates the point previously made regarding the significant influence of tissue properties. Accordingly, the present practice of rather unspecific stimulation of vastly different brain areas needs to be challenged.

Conversely, precision targeting will enable specific DBS interventions including different lead types, configurations and parameter selections depending on individual and regional neuroanatomical tissue properties (for interindividual differences in neural connectivity see Mueller et al., 2013, for consequences of large interindividual variability for human brain atlases see Uylings et al., 2005, and for a recent study on the differential effects of hyperdirect pathway vs. indirect pathway stimulation see Neumann et al., 2018). Finally, the properties of the tissue surrounding the electrodes may deliberately be used to achieve precision targeting during stimulation.

### Outlook: The Time Is Right for Individualized Precision Targeting and More Evidence-Based DBS Interventions on the Basis of More Accurate and Sophisticated Models

Although simplified in its execution, the presented work attempts to demonstrate the current distribution on a larger area of brain tissue. Results gained from such and more refined models could prove useful in finding optimal stimulation parameters and lead configurations, and thereby reducing current leakage into surrounding tissue. Albeit more sophisticated simulations have been performed previously, their transferability to the clinical setting is still lacking. While appropriately parameterized finite element models may be used to accurately capture the generated potential and the volume of the activated tissue, the spread of stimulation as a function of individual electrode placement and stimulation parameter settings need to be quantified with further computational models. Ultimately, such models may lead to increased therapeutic responses and a significant reduction in side effects. Therefore, more realistic models which implement biophysical properties in a more refined way and simulate in 3D (see Limitations) should be more actively promoted. As such, they may provide the basis for more individualized precision targeting and more evidence-based neuromodulation interventions, which would affect thousands of patients worldwide. This is not to say that computational models are the only necessary way to improve DBS interventions.

As outlined above, the unique location of the STN between large fiber-tracts (between the ic and, e.g., the fct), which influence the electric field cannot be applied to other targets. Accordingly, each DBS intervention should not only be individualized according to the general biophysical tissue properties of the target, but also according to the individual neuroanatomical specificities of the patient. A more individualized DBS approach has been advocated by many scholars. Such individualization may include the consideration of individual myelin-fiber tracts and preoperative functional neuroimaging (Gunalan et al., 2017). Electrode position and the necessary application energy may be precalculated in such a personalized approach. Since technological possibilities and academic knowledge are increasing steadily, both trial-and-error strategies and imitation practices are becoming less and less ethically justifiable.

Image-assisted personalized programming may also be applied for the calculation of the electrode’s rotation angle. The possibility of determining the exact orientation of the leads has recently been demonstrated with rotational 3D fluoroscopy (Reinacher et al., 2017). More accurate identification of the physical tissue properties (including permeability and conductivity) remains a challenge, especially if living whole brain tissue of human origin and not only isolated biological tissues are considered. In fact, in an isotropic finite element model, tissue properties are determined by scalar values, whereas in an anisotropic model, tensors (three dimensional matrices) are necessary to represent the ratio and orientation of the anisotropy. The anisotropic conductivity may be derived from DTI by measurement of the effective Brownian motion of water molecules applied to a variable magnetic field. This has already been achieved in 2005 by Butson and McIntyre (2005). In addition, more knowledge of biophysical interactions between the stimulation and neural elements is needed. Finally, the combination of simulations with *in-vivo* measurements, although performed in a preclinical setting, has already been demonstrated (Dmochowski and Bikson, 2017; Grossman et al., 2017).

Furthermore, the validation of targeting in deep brain regions is challenging, as imaging tools for these areas are scarce. While encephalography is characterized by poor spatial resolution, the blood-oxygenation level dependent signal (BOLD) in fMRI is difficult to apply to neural signaling. These imaging limitations underline the need for computer-based models to improve precision targeting. Ideally, automated algorithms may efficiently identify the optimal combination of electrode positioning and current intensities to precisely target the desired brain regions (Dmochowski et al., 2011). Future DBS leads should not only be characterized by a segmented nature, but also include the ability to measure the present electrical field expansion and thereby adapt to the local situation. Altogether, an effective model needs to take an interdisciplinary approach including knowledge from basic, preclinical and clinical research in order to effectively and ethically improve DBS interventions (Ineichen et al., 2014). Mere knowledge of the electric field distribution itself is insufficient, and as such the development of functional models is equally important.

So far, our simulation data has not been able to explain such important side effects as dysarthria after DBS (Sidtis and Sidtis, 2017), but it has the potential to do so. In the future, 3D modeling of electrical spread could help identify crucial impact on nuclei and fiber-tracts interacting in dysarthria development (cerebellum-midbrain nuclei-basal ganglia-thalamus). As shown in DBS for essential tremor, using an anatomic atlas to define the stimulation location could aid in optimizing speech outcome (Matsumoto et al., 2016).

### Limitations

The following limitations of the present study should be mentioned: In order to further enhance the model, tissue properties such as precise white and gray matter conductivities (e.g., from DTI data similar to Butson and McIntyre, 2005) should be implemented. In order to achieve this, the cytoarchitectonic and microscopic neuroanatomic diversity of the human brain should be isolated. Previous research has furthermore highlighted how the electrode potential is influenced by brain pulsation (Yousif et al., 2007). The electrode potential is also affected by hydration state, which is particularly important in older patients (for example in patients suffering from PD), who often suffer from reduced water intake (exsiccosis). Glial scar formation around the electrode (Howell et al., 2015) also influences current spread. The circumstances mentioned here have not been accommodated in the presented model.

Local fluid retention (Åström et al., 2006), usually an acute reaction after electrode implantation, has not been regarded in this model. The distinction between acute and chronic stages (see Yousif et al., 2007: shunting and shielding effects) has also been mentioned. Moreover, the shape and extent of the electric field created by electrode activation is modulated by physiological as well as pathophysiological factors, such as the above-mentioned glial scar formation.

In sum, this work is far from complete and cannot claim to be a fully accurate depiction of electrical field spread. Rather, it provides an exemplary basis for continuing important work that focuses on electric field generation in the brain tissue. It also seeks to actively stress the importance of further contributions as the foundation for improvement of an intervention used for thousands of patients worldwide. This paper aims to promote the development of DBS from an *ad-hoc* medical intervention to a more knowledge-guided, evidence-based approach.

## Conclusion

The present work emphasizes the generally held view that brain tissue properties have a significant influence on electric field distribution during DBS. The results of the presented simulation as well as earlier data encourage us to conclude that the current DBS intervention practice needs to be adapted and improved. Selective parameters such as frequency, intensity and (ultra-short) pulse width, for example, need to be adjusted with higher precision and according to individual conditions.

This work introduces the amazingly powerful effects of biophysical tissue properties on the electric field over a larger area of tissue. While the use of segmented electrodes supports a better regulation of the electric current when compared to omnidirectional electrodes, successful DBS treatment also relies heavily on accurate image-based targeting. Also worthy of consideration is rotation of the segmented electrode toward functional areas intended for stimulation.

Better targeting may be achieved with validated and more refined models, sophisticated hardware options and better visualization options. The adaptation of the modeling and simulation to the individual neuroanatomy may lead to a reduction in side-effects caused by current leakage. Furthermore, the therapeutic effects may be increased by higher specificity. Improved DBS leads should be equipped to measure local electrical field expansion and allow for specific adjustments of the electric current. As “smart” technology spreads in every domain of our lives, patients’ physical and mental activity may be recorded, and consecutive electrical stimulation may be adapted accordingly.

In conclusion, we have enriched the evidence that dielectric structures surrounding a small neuronal target largely determine the resulting electrical spread. Before the era of DBS, stereotactic lesions generated with heat appeared in spherical or ellipsoid forms. It is unwise, however, to simplify electrical field spread to this archaic level. The application of electrode configurations and parameter settings, often chosen irrespective of tissue properties at stimulation sites for various neuronal areas, appears to be applied in a universally similar fashion. This proves that a transfer from computational models to clinical practice has yet to be achieved. The current practice also reflects the mistaken yet persistent notion of presumably many clinicians regarding electrical field distribution that neglects the influence of tissue inhomogeneity and anisotropy. Nevertheless, the spread of electric field also depends on lead type, placement, rotation and stimulation properties. After three decades of research, the implementation of more sophisticated biophysical models with clinical transferability for DBS is long overdue.

## Author Contributions

CI performed the calculations, analysis, and visualizations of the field-modeling. OS provided anatomical and neurosurgical expertise for visualizations and clinical implications. CI, NS, and OS wrote the paper. CI furthermore confirms that he has final responsibility for the decision to submit for publication.

## Conflict of Interest Statement

The authors declare that the research was conducted in the absence of any commercial or financial relationships that could be construed as a potential conflict of interest.

## Abbreviations

al: ansa lenticularis
ac: anterior commissure
bic: brachium of the inferior colliculus
Cd: caudate nucleus
CT: computer tomography
DBS: deep brain stimulation
DTI: diffusion tensor imaging
fct: fasciculus cerebello-thalamicus
FEM: finite element method
fx: fornix
GPi and GPe: respectively, globus pallidus internus and externus
Hyp: hypothalamus
ic: internal capsule
MRI: magnetic resonance imaging
mtt: mammillothalamic tract
MGN and LGN: respectively, medial and lateral geniculate nucleus
ml: medial lemniscus
Acb: nucleus accumbens
PDEs: partial differential equations
PAG: periaqueductal (or central) gray
pc: posterior commissure
Pul: pulvinar
Put: putamen
R: reticular thalamic nucleus
RN: red nucleus
STh or STN: subthalamic nucleus
